# Regulatory T cells in psoriatic arthritis: an IL-17A-producing, Foxp3^int^CD161 + RORγt + ICOS + phenotype, that associates with the presence of ADAMTSL5 autoantibodies

**DOI:** 10.1038/s41598-022-24924-w

**Published:** 2022-11-30

**Authors:** Juliëtte N. Pouw, Michel A. M. Olde Nordkamp, Tessa van Kempen, Arno N. Concepcion, Jacob M. van Laar, Femke van Wijk, Julia Spierings, Emmerik F. A. Leijten, Marianne Boes

**Affiliations:** 1 Department of Rheumatology and Clinical Immunology, University Medical Center Utrecht, Utrecht University, H03.103, P.O. Box 85500, 3508 GA Utrecht, The Netherlands; 2 Center for Translational Immunology, University Medical Center Utrecht, Utrecht University, 3508 AB Utrecht, The Netherlands; 3 Biomedical Laboratory Sciences, Avans University of Applied Sciences, 4800 RA Breda, The Netherlands; 4 Department of Rheumatology, Sint Maartenskliniek, 6500 GM Nijmegen, The Netherlands; 5 Department of Pediatric Immunology, Wilhelmina Children’s Hospital, Utrecht University, 3508 AB Utrecht, The Netherlands

**Keywords:** Immunology, Rheumatology

## Abstract

In psoriatic arthritis (PsA), predisposing class I HLA alleles, the presence of synovial clonally proliferated CD8 + T cells and autoantibodies all point towards the loss of immune tolerance. However, the key mechanisms that lead to immune dysregulation are not fully understood. In other types of inflammatory arthritis, T regulatory cell (Treg) dysfunction and plasticity at sites of inflammation were suggested to negatively affect peripheral tolerance. We here addressed if Treg variances associate with psoriatic disease. We collected clinical data, sera and peripheral blood mononuclear cells from 13 healthy controls, 21 psoriasis and 21 PsA patients. In addition, we obtained synovial fluid mononuclear cells from 6 PsA patients. We studied characteristics of CD4 + CD25 + CD127^lo^Foxp3 + Tregs by flow cytometry and used ELISA to quantify antibodies against ADAMTSL5, a recently discovered autoantigen in psoriatic disease. In comparison with their circulating counterparts, Tregs from inflamed joints express increased levels of ICOS, CTLA-4 and TIGIT. Furthermore, synovial fluid-derived Tregs have a distinct phenotype, characterized by IL-17A production and upregulation of CD161 and RORγt. We identified a subset of Tregs with intermediate Foxp3 expression as the major cytokine producer. Furthermore, ICOS + Tregs associate with PsA disease activity as measured by PASDAS. Lastly, we observed that presence of the Foxp3^int^ Tregs associates with an increased abundance of anti-ADAMTSL5 autoantibodies. Tregs derived from the inflammatory environment of inflamed PsA joints exhibit a distinct phenotype, which associates with loss of peripheral immune tolerance in psoriatic disease.

## Introduction

Psoriatic arthritis (PsA) is a heterogeneous, inflammatory, musculoskeletal disease characterized by psoriasis, arthritis, enthesitis, dactylitis and nail dystrophy. PsA is the second most common type of inflammatory arthritis and develops in up to 30% of patients with psoriasis^1^. Increasing evidence suggests that autoimmune mechanisms underlie PsA pathogenesis, including strong associations with class I human leukocyte antigen alleles, ectopic lymphoid neogenesis in synovial tissues with T and B cell aggregates, presence of autoantibodies, and clonally proliferated CD8 + T cells in synovial tissue and fluid^2–4^. However, the key immunological factors that decrease immune tolerance and lead to PsA transition in psoriasis patients remain largely unknown^1,5^.

In auto-immune rheumatic diseases, regulatory T cells (Tregs) derived from synovial fluid were shown to effectively suppress effector T cells and thus maintain immune homeostasis^6,7^. However, other studies implicated Tregs in a pathogenic role, showing data that loss of peripheral immune tolerance associated with impaired expression of key immune regulatory molecules and through Treg differentiation^8–11^. Tregs in an inflammatory micro-environment may differentiate under the influence of T cell receptor engagement, IL-2 deprivation and pro-inflammatory cytokines^10,12,13^. Consequently, Tregs can downregulate their key transcription factor forkhead box P3 (Foxp3) and obtain effector T cell (Teff) phenotype and function^11,13^. Moreover, Treg differentiation–or plasticity–can be accompanied by reduced suppressive function, expression of T helper 17 cell features (CD161, retinoic acid-related orphan receptor gamma t (RORγt)) and production of pro-inflammatory cytokines^8,11,13–17^. Hence, these results suggest that differentiated Tregs in inflammatory arthritis may become pathogenic and amplify inflammation, instead of halting disease^18,19^.

With regards to Tregs in patients with psoriatic disease, literature is scarce. Treg abnormalities have been observed, mostly in patients with psoriasis, including decreased expression of CD39 and CD74^20^, increased expression of IL-6Rα^21^, reduced suppressive capacity^15,22–24^, chemotactic deficiency^25^ and the enhanced propensity to differentiate into cells that produce interleukin (IL)-17–the hallmark cytokine of psoriatic disease^15^. Only few studies investigated the role of Tregs in PsA pathogenesis and in-depth characterization of intra-articular Tregs is lacking^26^. With this study we aimed to study a possible role for Treg phenotypical variances in PsA pathogenesis and loss of peripheral tolerance.

## Methods

### Study design

We performed an observational cohort study at the University Medical Center Utrecht, the Netherlands. The study protocol was approved by the medical research ethics committee Utrecht (protocol number 13-696). We obtained written informed consent from all participants. The work has been carried out in accordance with The Code of Ethics of the World Medical Association (Declaration of Helsinki).

### Subjects

We included patients aged 18 years or older with a diagnosis of psoriasis or PsA. PsA patients met the ‘ClASsification for Psoriatic ARthritis’ (CASPAR) criteria. We defined psoriasis as a confirmed diagnosis of psoriasis and absence of inflammatory arthritis. The latter was assessed by medical history, physical examination and laboratory parameters. We excluded patients that used disease-modifying anti-rheumatic drugs (DMARDs) in the past three months. In addition, we collected synovial fluid from patients with a clinical diagnosis of PsA, gout and osteoarthritis (OA).

### Disease activity

To quantify disease activity we used two validated, disease-specific composite measures for PsA: Disease Activity index for PSA (DAPSA)(range 0–164) and Psoriatic ArthritiS Disease Activity Score (PASDAS)(range 0–10). We used patient-reported outcomes (PROs) to assess disease severity with six questionnaires: dermatology life quality index, health assessment questionnaire, short form-36 physical and mental component score, visual analogue scale for pain and patient global assessment.

### Samples

We performed cross-sectional sampling of peripheral blood and synovial fluid. To collect sera, we centrifuged BD Vacutainer™ Plastic Blood Collection serum Tubes (silica and polymer gel) for 10 min (1500 g, room temperature). We collected peripheral blood in BD Vacutainer™ Plastic Blood Collection Tubes with Lithium Heparin. For synovial fluid and synovial fluid mononuclear cells (SFMC), we obtained intra-articular fluid of swollen joints in sterile 10–50 mL syringes. We isolated SF by centrifugation for 10 min (2300 g). To isolate PBMC and SFMC we performed 25 min density centrifugation (400 g, Ficoll-Paque). We stored samples at −80 °C (sera, synovial fluid) and liquid nitrogen (PBMC, SFMC) until measurement.

### T cell activation assay

To assess Treg cytokine production upon activation, we cultured PBMC and SFMC in complete medium (RPMI 1640 GlutaMAX (61,870,044; Thermo Fisher Scientific) + 10% fetal bovine serum + 1% Penicillin–Streptomycin) with 20 ng/mL PMA (P8139-1MG, Sigma) and 1 µg/ml ionomycin (407,952, Calbiochem / EMD Chemicals inc.) for 4.5 h, while inhibiting protein transport with 1:1000 BD GolgiStop (51-2092KZ, BD Bioscience).

### Flow cytometry

We stained samples by incubation with 25 µl antibody mix diluted in buffer (500 ml phosphate-buffered saline + 5 ml 10% sodium azide + 5 g bovine serum albumin) for 25 min at 4 °C. Before intracellular stains, we fixed and permeabilized cells with 100 µl Fixation/Permeabilization Concentrate and Diluent (00–5123-43, 00–5223-56, eBioscience). Details of flow cytometry antibodies of both panels used are listed in Supplemental Table S1. Using fluorescence minus one (FMO) controls, we identified viable (assessed by a Fixable Viability Dye) CD3 + CD4 + CD25 + CD127^lo^Foxp3 + Tregs. To further study the phenotypical and functional properaties of Tregs with reduced expression of Foxp3, we studied Tregs with intermediate (Foxp3^int^) and high (Foxp3^hi^) Foxp3 expression. Importantly, we do not propose to have identified two different subsets of Tregs, but use this exploratory dichotomization of the Treg population to enable studying the differences between Tregs with high and intermediate Foxp3 expression. Of these Foxp3^int^ and Foxp3^hi^ Treg subsets, we used FMO controls to assess median fluorescent intensity (MFI) and proportions of cell populations that express cytotoxic T-lymphocyte-associated protein 4 (CTLA-4 = CD152), CD161, inducible T-cell costimulator (ICOS = CD278), T cell immunoreceptor with Ig and ITIM domains (TIGIT), Ki67 and RORγt. In addition to FMO controls, we found that the modest expression of RORγt and CD161 required standardization by using a uniform gate based on a representative healthy control sample of CD3 + CD4 + CD25 + CD127^lo^ Tregs. We standardized quantification of intracellular IL-10 and IL-17A by applying a cutoff value of < 0.5% in the medium control samples. Negative controls of flow cytometry analyses are shown in Supplemental Figure S1 and S2. We performed acquisition on the BD LSRFortessa (405, 488, 561, 635 nm lasers) with BD FACSDIVA (version 8.0.1). We used FlowJo (version 10.7.1) for further analyses.

### ELISA

We coated 96-well flat-bottom Nunc MaxiSorp™ plates (44–2404-21, ThermoFisher) overnight with 50 µL/well 5 µg/mL recombinant ADAMTSL5 peptide (NBP1-93438PEP, Novus Biologicals), diluted in 2% bovine serum albumin (BSA)(10,735,094,001, Roche) in phosphate buffered saline (PBS). Next, we blocked nonspecific binding sites for 1 h at room temperature with 100 µL/well 4% BSA in PBS and incubated overnight with 50 µL serially diluted patient serum or SF in duplo in 2% BSA in PBS. For the standard curve, we used 50 µL primary anti-human-ADAMTSL5 antibodies in duplo (HPA044050-100UL, Sigma-Aldrich), serially diluted in 2% BSA in PBS in the following concentrations: 5.00, 1.67, 0.56, 0.1852, 0.0617, 0.0206, 0.0069 μg/mL. After overnight incubation at 4°C, we incubated patient sample wells with 50 µL/well horseradisch-peroxidase (HRP)-conjugated anti-human IgG (11,869,130, ThermoFisher Scientific) and standard curve wells with 50 µL HRP-conjugated anti-rabbit IgG (31,460, Thermofisher). We developed and stopped the color reaction with 50 µL/well of 3,3′, 5,5′-tetramethylbenzidine (TMBW-1000–01, Tebu-Bio) and 2 N H_2_SO_4_, respectively. We measured absorbance at 450 nm with a reference wavelength of 570 nm. We selected the dilution that best fitted the 5-parameter fit curve using software by Clariostar (version 5.40 R2; firmware version 1.2; serial number 430–1031) and MARS (version 3.31).

### Statistical analysis

We applied Wilcoxon Singed Rank tests to compare characteristics between Foxp3^int^ and Foxp3^hi^ Tregs. To compare flow cytometry and ELISA results between patient groups, we used Mann–Whitney U (MWU) tests. Synovial fluid-derived Tregs were only compared with peripheral blood Tregs from PsA patients. To test the association of clinical outcomes, Treg characteristics and autoantibody concentration, we used Spearman's rank correlation. We performed contingency analyses using χ^2^ tests for categorical variables, and independent samples T-tests or MWU tests for continuous variables, to analyze patient clinical characteristics. A P-value of < 0.05 was considered statistically significant. Statistical analyses were performed with IBM SPSS statistics (Version 26 Release 26.0.0.1) and GraphPad Prism 8 (Version 8.3.0).

### Ethics approval and consent to participate

The study protocol was approved by the medical research ethics committee Utrecht (protocol number 13-696). We obtained written informed consent from all participants.

## Results

### Cohort

**Figure 1:**
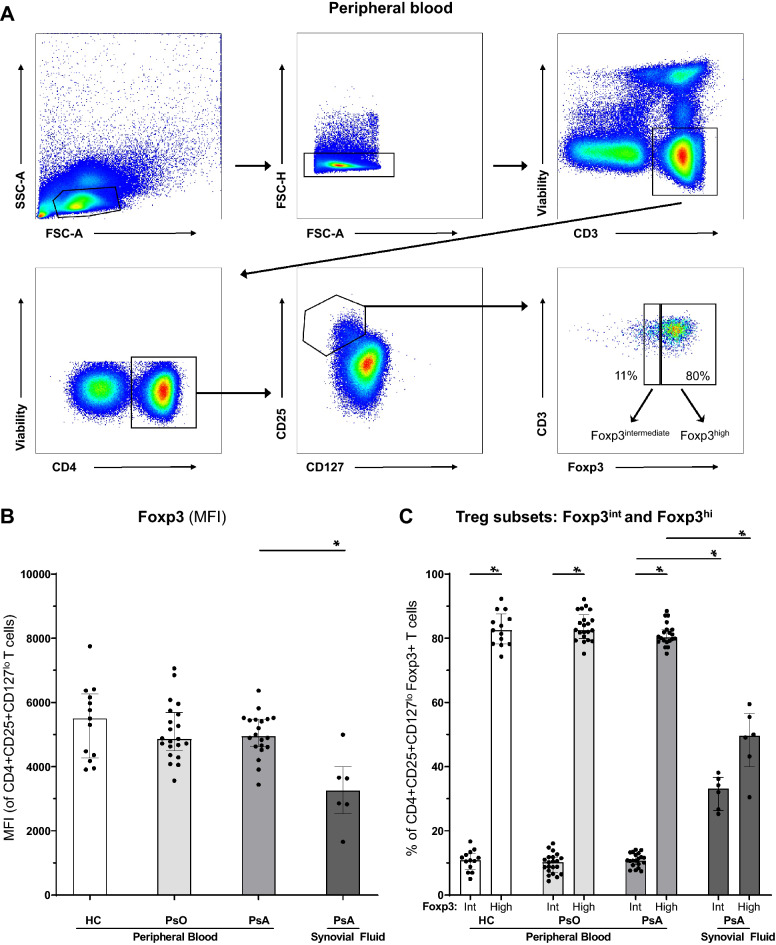
Increase of Tregs with intermediate Foxp3 expression in inflamed PsA joints. Flow cytometry analysis of Foxp3 expression by CD4 + CD25 + CD127^lo^ T cells derived from peripheral blood of HC (n = 13), psoriasis patients (n = 21) and PsA patients (n = 20), and from synovial fluid of PsA patients (n = 6). Bar graphs: symbols represent individual subjects; bars show median with interquartile range; *P value < 0.05 (PsA synovial fluid only compared with PsA peripheral blood). (**A**) Gating strategy for CD4 + CD25 + CD127^lo^Foxp3 + Tregs. We differentiated between Tregs with intermediate and high expression of Foxp3 (Foxp3^int^ and Foxp3^hi^, respectively). Percentages in dot plots represent median of peripheral blood-derived Tregs in PsA. (**B**) Foxp3 expression by CD4 + CD25 + CD127^lo^ T cells. (**C**) Proportion of Foxp3^int^ and Foxp3^hi^ Tregs of CD4 + CD25 + CD127^lo^ T cells. Foxp3^int^/^hi^: forkhead box P3 expression intermediate / high; FSC-A: forward scatter area; FSC-H: forward scatter height; HC: healthy control; MFI: median fluorescent intensity; PsA: psoriatic arthritis; PsO: psoriasis; SSC-A: sideward scatter area; Treg: T regulatory cell.

To investigate Treg phenotypical variances in psoriatic disease, we studied characteristics of Tregs in peripheral blood from 13 healthy controls (HC), 21 psoriasis patients and 21 PsA patients, and in synovial fluid of 6 PsA patients. Detailed patient characteristics are shown in Supplemental Table S2. We identified Tregs using the best available discriminative markers: CD3 + CD4 + CD25 + CD127^lo^Foxp3 + (Fig. 1A)^18,19,27,28^.

### Increase of CD4 + CD25 + CD127^lo^Foxp3 + tregs with intermediate Foxp3 expression in PsA synovial fluid

We observed that synovial fluid-derived Tregs, as compared to peripheral blood, have lower expression of Foxp3 (MFI 3248 vs. 4948, P = 0.002)(Fig. 1B). As Foxp3 is the key transcription factor of Treg development, maintenance and function, this finding raised our interest in phenotypical and functional properties of Tregs with reduced expression of Foxp3^29^. Therefore, we studied two subset of Tregs: with intermediate (Foxp3^int^) and high (Foxp3^hi^) Foxp3 expression (Fig. 1C). CD4 + CD25 + CD127^lo^ T cells without Foxp3 were excluded from further analyses. As compared to circulating T cells from PsA patients, in synovial fluid we observed an increase of Foxp3^int^ Tregs of CD3 + CD4 + CD25 + CD127^lo^ lymphocytes from 11 to 33% in SF (*P* < 0.001) and a decrease of Foxp3^hi^ Tregs from 80 to 50% (*P* < 0.001). The percentages of Foxp3^int^ and Foxp3^hi^ Tregs were strongly negatively correlated in both PBMC and SFMC of HC, PsO and PsA patients (ρ > 0.9, *P* < 0.05)(Supplemental Figure S3). We observed no significant differences in Foxp3 expression between healthy controls, psoriasis and PsA patients.

### Ki67-expressing tregs are increased in inflamed PsA joints

Subsequently, we focused on differences between Foxp3^int^ and Foxp3^hi^ Tregs in peripheral blood and synovial fluid by assessing their relative frequencies and proliferative capacity. Compared to peripheral blood, synovial fluid was significantly enriched for Foxp3^int^ Tregs (*P* < 0.001)(Supplemental Figure S4A and S4B). When examining the proliferative capacity, we noted that in general Foxp3^hi^ Tregs had higher proliferative capacity than Foxp^int^ Tregs. Nonetheless, both subsets (Foxp^int^ and Foxp3^hi^) derived from synovial fluid had higher proliferative capacity compared to their peripheral blood counterparts (Supplemental Figure S4C-E).

### A subset of tregs in inflamed joints in PsA upregulate CD161 and RORγt

**Figure 2:**
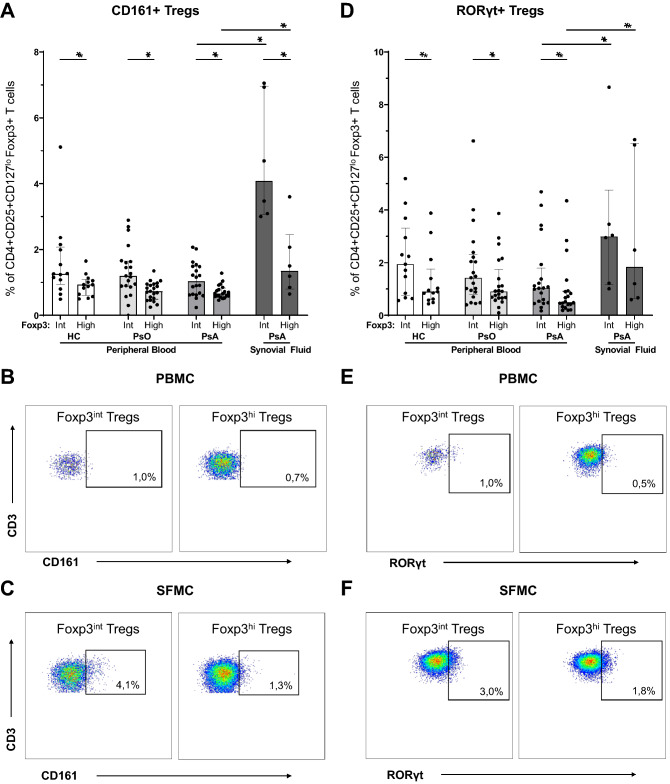
CD161 + RORγt + phenotype of synovial fluid-derived Foxp^int^ and Foxp3^hi^ Tregs. Flow cytometry analysis of CD4 + CD25 + CD127^lo^Foxp3 + Tregs derived from peripheral blood of HC (n = 13), psoriasis patients (n = 21) and PsA patients (n = 20), and from synovial fluid of PsA patients (n = 6). Bar graphs: symbols represent individual subjects; bars show median with interquartile range; *P value < 0.05 (synovial fluid only compared with PsA peripheral blood). Dot plots: percentages in PBMC plots represent median of PB-derived Tregs in PsA; percentages in SFMC plots represent median of SF-derived Tregs. (**A**) Proportions of CD161 + Foxp3^int^ and CD161 + Foxp3^hi^ Tregs. (**B-C**) Representative flow cytometry plots to identify CD161 + Foxp3^int^ and -Foxp3^hi^ Tregs derived from peripheral blood (B) and synovial fluid (C). (**D**) Proportions of RORγt + Foxp3^int^ and RORγt + Foxp3^hi^ Tregs. (**E–F**) Representative flow cytometry plots to identify RORγt + Foxp3^int^ and -Foxp3^hi^ Tregs derived from peripheral blood (E) and synovial fluid (F). Foxp3^int/-hi^: forkhead box P3 expression intermediate/high; HC: healthy control; PBMC: peripheral blood mononuclear cells; PsA: psoriatic arthritis; PsO: psoriasis; RORγt: retinoic acid receptor-related orphan receptor gamma; SFMC: synovial fluid mononuclear cells; Tregs: T regulatory cells.

To further investigate the phenotype of Foxp3^int^ and Foxp3^hi^ synovial fluid-derived Tregs in PsA we studied CD161 and RORγt, as they are associated with arthritis and a pro-inflammatory potential of Tregs^7,16,30^. In PsA patients, we found that the percentage of CD161-expressing Tregs was higher in synovial fluid than in circulation and that more Foxp3^int^ Tregs express CD161 (4.1%), as compared to Foxp3^hi^ Tregs (1.3%)(Fig. 2A–C). Additionally, in PsA, synovial fluid Tregs express more RORγt than Tregs in circulation (Foxp3^int^ 3.0 vs. 1.0%, P = 0.048; Foxp3^hi^ 1.8% vs. 0.5% Treg, P = 0.026)(Fig. 2D–F). The increased expression of CD161 and RORγt by synovial Tregs was most pronounced in the intermediate Foxp3 subset of Tregs (Fig. 2A and D). Again, no differences were found between peripheral blood Tregs of healthy controls, psoriasis and PsA patients.

### High IL-10 and IL-17A production by synovial fluid-derived tregs

**Figure 3:**
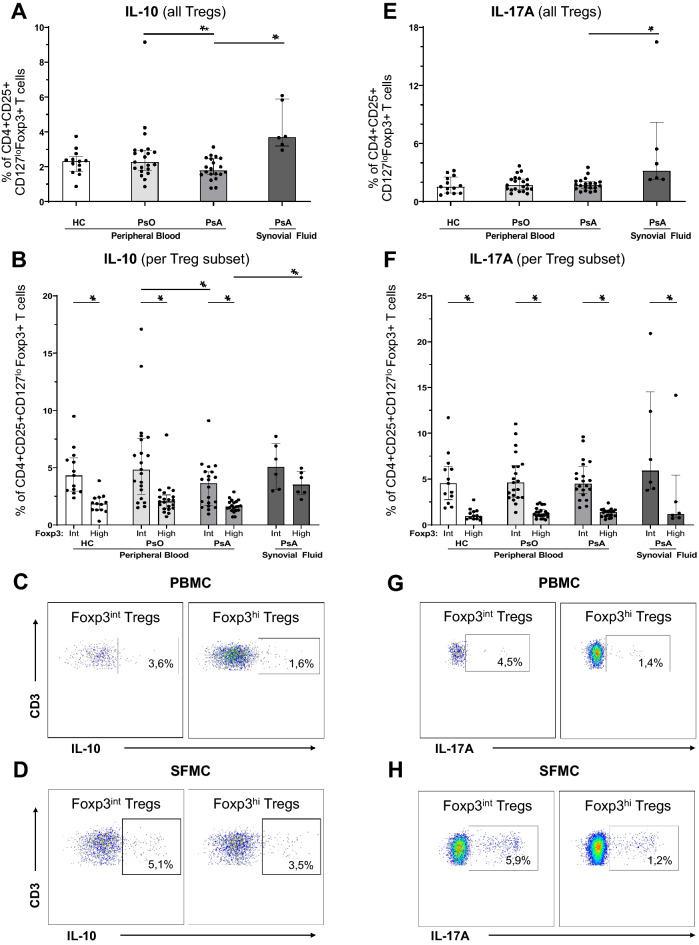
IL-10 and IL-17A production by intra-articular Tregs in PsA patients. Flow cytometry analysis of CD4 + CD25 + CD127^lo^Foxp3 + Tregs derived from peripheral blood of HC (n = 13), psoriasis patients (n = 21) and PsA patients (n = 20), and from synovial fluid of PsA patients (n = 6). PBMC and SFMC were cultured for 4,5 h with 20 ng/mL PMA, 1 µg/ml ionomycin and 1:1000 BD GolgiStop. Bar graphs: symbols represent individual subjects; bars show median with interquartile range; *P value < 0.05 (synovial fluid only compared with PsA peripheral blood). Dot plots: percentages in PBMC plots represent median of PB-derived Tregs in PsA; percentages in SFMC plots represent median of SF-derived Tregs. (**A**) Proportion of CD4 + CD25 + CD127^lo^Foxp3 + Tregs, that upon activation produce IL-10. (**B**) Proportions of Foxp3^int^ and Foxp3^hi^ Tregs, that upon activation produce IL-10. (**C**–**D**) Representative flow cytometry plots to identify IL-10 production by Foxp3^int^ and Foxp3^hi^ Tregs derived from peripheral blood (C) and synovial fluid (D). (**E**) Proportion of CD4 + CD25 + CD127^lo^Foxp3 + Tregs, that upon activation produce IL-17A. (**F**) Proportions of Foxp3^int^ and Foxp3^hi^ Tregs, that upon activation produce IL-17A. (**G**–**H**) Representative flow cytometry plots to identify IL-17A production by Foxp3^int^ and Foxp3^hi^ Tregs derived from peripheral blood (G) and synovial fluid (H). Foxp3^int^/^-hi^: forkhead box P3 expression intermediate / high; HC: healthy control; IL: interleukin; PBMC: peripheral blood mononuclear cells; PsA: psoriatic arthritis; PsO: psoriasis; SFMC: synovial fluid mononuclear cells; Tregs: T regulatory cells.

To study functional differences between Tregs in an inflammatory environment and in circulation of PsA patients, we measured inhibitory and pro-inflammatory cytokine production. As expected, Tregs from synovial fluid showed a modest but increased capacity to produce cytokines, both the anti-inflammatory cytokine IL-10 (3.7% vs. 1.8%, *P* < 0.001)(Fig. 3A) and the key pro-inflammatory cytokine IL-17A (3.2% vs. 1.7%; P = 0.002) (Fig. 3E). Moreover, Tregs from PsA patients produce less IL-10, as compared to psoriasis patients (Fig. 3A and B). When examining the different subsets of Tregs, we found that the Foxp3^int^ subset was the major cytokine producer, the most notable being the elevated IL-17A producing capacity by Foxp3^int^ synovial fluid Tregs (5.9% vs. 1.2%, P = 0.028)(Fig. 3B-D and 3F-H). We observed that—both in peripheral blood and in synovial fluid—PsA patients have a higher proportion of single-IL-17A cytokine-producing Tregs as compared to healthy controls and psoriasis patients. A minority of Tregs produce both IL-10 and IL-17A (Supplemental Figure S5).

### Synovial fluid-derived tregs express high CTLA-4, TIGIT and ICOS

**Figure 4:**
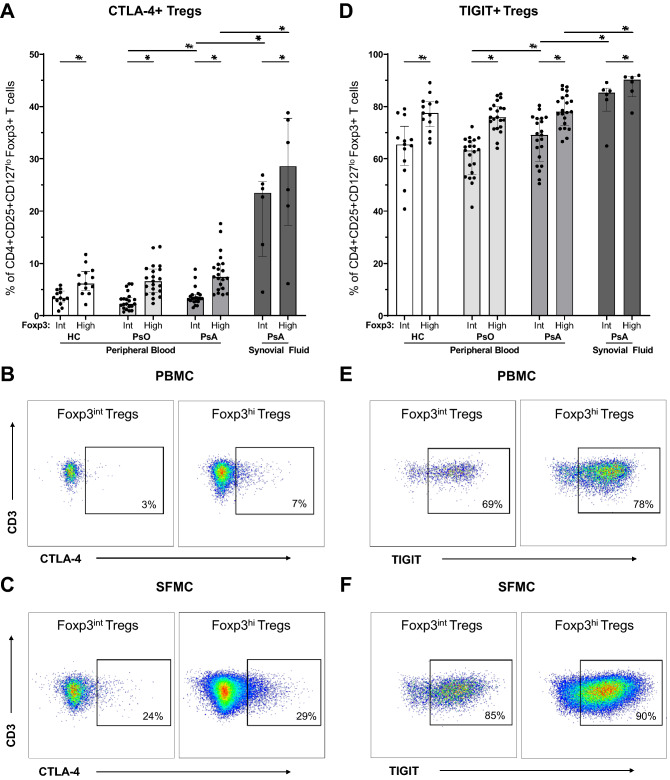
High expression of key regulatory immune receptors by intra-articular Tregs in PsA. Flow cytometry analysis of CD4 + CD25 + CD127^lo^Foxp3 + Tregs derived from peripheral blood of HC (n = 13), psoriasis patients (n = 21) and PsA patients (n = 21), and from synovial fluid of PsA patients (n = 6). Bar graphs: symbols represent individual subjects; bars show median with interquartile range; *P value < 0.05 (synovial fluid only compared with PsA peripheral blood). Dot plots: percentages in PBMC plots represent median of PB-derived Tregs in PsA; percentages in SFMC plots represent median of SF-derived Tregs. (**A**) Proportion of CTLA-4 + Foxp3^int^ and CTLA-4 + Foxp3^hi^ Tregs. (**B,C**) Representative flow cytometry plots to identify CTLA-4 + Foxp3^int^ and -Foxp3^hi^ Tregs derived from peripheral blood (**B**) and synovial fluid (**C**). (**D**) Proportions of TIGIT + Foxp3^int^ and TIGIT + Foxp3^hi^ Tregs. (**E,F**) Representative flow cytometry plots to identify TIGIT + Foxp3^int^ and -Foxp3^hi^ Tregs derived from peripheral blood (**E**) and synovial fluid (**F**). CTLA-4: cytotoxic T-lymphocyte-associated protein 4 (CD152); Foxp3^int^/^-hi^: forkhead box P3 expression intermediate / high; HC: healthy control; PBMC: peripheral blood mononuclear cells; PsA: psoriatic arthritis; PsO: psoriasis; SFMC: synovial fluid mononuclear cells; TIGIT: T cell immunoreceptor with Ig and ITIM domains; Tregs: T regulatory cells.

Reduced expression of immune receptors by Tregs could contribute to abnormal Treg function in inflammatory arthritis^8^. Therefore, we measured expression of two key inhibitory receptors essential for Treg suppressive function: CTLA-4 and TIGIT (Fig. 4A–F). Both receptors were expressed more by Foxp3^hi^ Tregs, as compared to the intermediate Treg subset (Fig. 4A and D). In PsA patients, the proportions Tregs that express CTLA-4 were increased in synovial fluid, as compared to peripheral blood (Foxp3^int^ 23.5% vs. 3.3%, P = 0.000; Foxp3^hi^ 28.6% vs. 7.4%, P = 0.003)(Fig. 4A–C). That was similar for TIGIT: proportions of Foxp3^int^ and Foxp3^hi^ Tregs with TIGIT expression were higher in synovial fluid (Foxp3^int^ 85.3% vs. 69.1%, P = 0.002; Foxp3^h^: 90.3% vs. 78.0%, P = 0.004)(Fig. 4D–F). Moreover, we observed higher CTLA-4 and TIGIT expression by Foxp3^int^ Tregs of PsA patients as compared to psoriasis patients (CTLA-4 + Tregs: 2.3% vs. 3.3%, P= 0.040)(TIGIT + Tregs: 63.2% vs. 69.1%, P = 0.017)(Fig. 4A and B).

**Figure 5:**
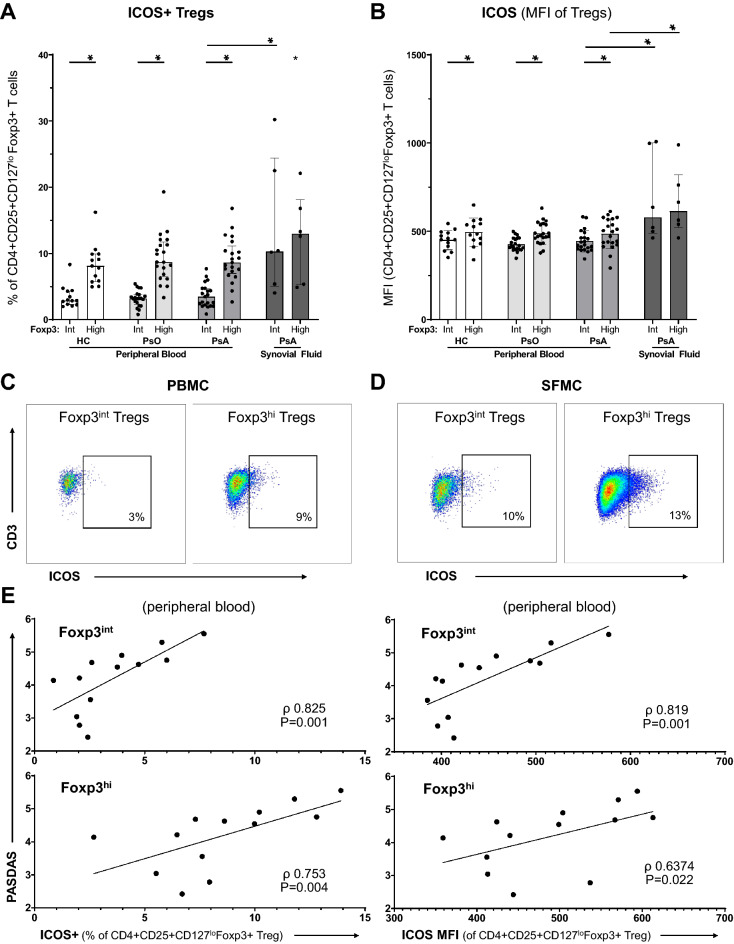
High PsA disease activity associates with ICOS + Tregs in circulation. Flow cytometry analysis of CD4 + CD25 + CD127^lo^Foxp3 + Tregs derived from peripheral blood of HC (n = 13), psoriasis patients (n = 21) and PsA patients (n = 21), and from synovial fluid of PsA patients (n = 6). Bar graphs: symbols represent individual subjects; bars show median with interquartile range; *P value < 0.05 (synovial fluid only compared with PsA peripheral blood). (**A**) Proportions of ICOS + Foxp3^int^ and ICOS + Foxp3^hi^ Tregs. (**B**) ICOS expression by Foxp3^int^ and Foxp3^hi^ Tregs as measured by MFI. (**C-D**) Representative flow cytometry plots to identify ICOS + Foxp3^int^ and -Foxp3^hi^ Tregs derived from peripheral blood (**C**) and synovial fluid (**D**). Percentages in PBMC dot plots represent median of PB-derived Tregs in PsA. Percentages in SFMC dot plots represent median of SF-derived Tregs. (**E**) Scatterplots of significant Spearman’s rank correlation of disease activity of PsA (as measured by PASDAS (range 0–10)) with ICOS expression by CD4 + CD25 + CD127^lo^Foxp3 + Tregs. Association shown of PASDAS with the proportion of ICOS + Foxp3^int^ Tregs (upper left), proportion of ICOS + Foxp3^hi^ Tregs (lower left), ICOS expression as measured by MFI of Foxp^int^ Tregs (upper right) and ICOS expression as measured by MFI of Foxp^hi^ Tregs (lower right). CI: confidence interval; Foxp3^int^/^-hi^: forkhead box P3 expression intermediate / high; HC: healthy control; ICOS: inducible T-cell costimulator (CD278); MFI: median fluorescent intensity; PASDAS: psoriatic arthritis disease activity score; PBMC: peripheral blood mononuclear cells; PsA: psoriatic arthritis; PsO: psoriasis; ρ: Spearman’s rho; SFMC: synovial fluid mononuclear cells; Tregs: T regulatory cells.

Moreover, we included ICOS in our phenotypical Treg characterization, because ICOS + Tregs can play a pro-inflammatory, pathogenic role in inflammatory arthritis and immune diseases^31,32^. We observed a comparable expression pattern as for the inhibitory receptors: synovial fluid derived Tregs express more ICOS, as compared to Tregs in circulation (Fig. 5A–D). Furthermore, we found a difference between the Foxp3^int^ and Foxp3^hi^ Treg subsets: in peripheral blood Foxp3^int^ Tregs express less ICOS as compared to Foxp3^hi^ (3.5% vs. 8.6%, P = 0.000), but in synovial fluid both subset express similar levels (10.3% vs. 13.0%, P = 0.753)(Fig. 5A). This is a relevant finding, because we observed an association of ICOS expression on Treg with PsA disease activity as measured by PASDAS (range 0–10), which takes arthritis, enthesitis, dactylitis, C-reactive protein, physician disease activity score and two PROs into account (Fig. 5E). Both the proportion of ICOS + Tregs and the MFI of ICOS significantly correlated with PASDAS in the Foxp3^int^ Treg subset and the Foxp3^hi^ Treg subset. No significant differences were found between healthy controls, psoriasis and PsA patients.

### ADAMTSL5 autoantibodies associate with treg Foxp3 expression in psoriatic disease

**Figure 6:**
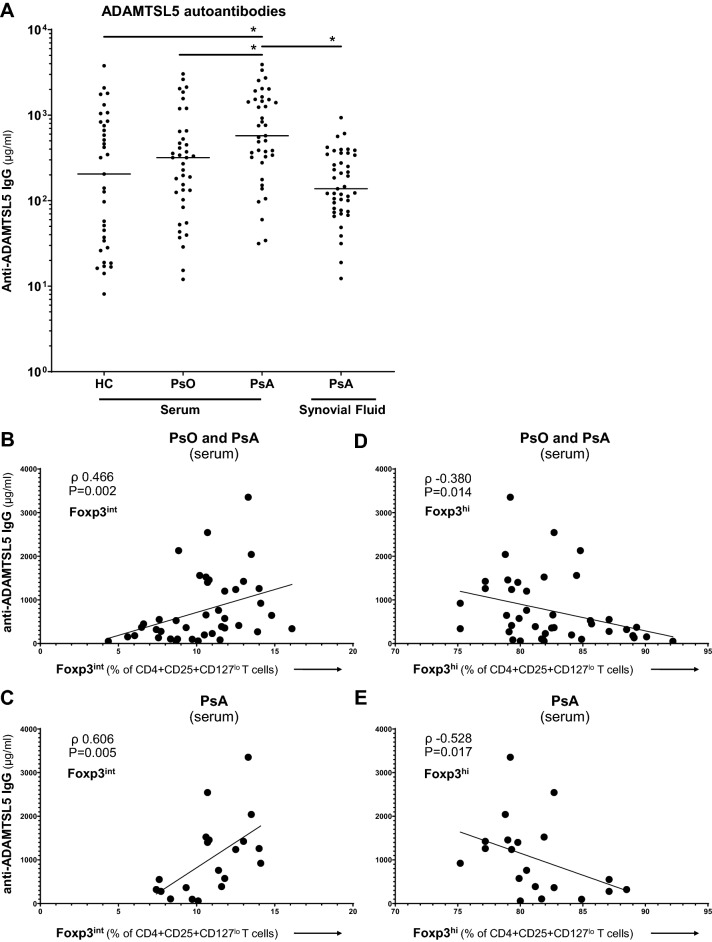
Foxp3 downregulation by Tregs associates with ADAMTSL5 autoantibodies in psoriatic disease. Association of ADAMTSL5 autoantibodies as measured by ELISA with Foxp3 expression by Tregs as measured by flow cytometry. (**A**) ELISA of anti-ADAMTSL5 IgG in sera of HC (n = 35), psoriasis patients (n = 39), PsA patients (n = 39), and synovial fluid of PsA patients (n = 43). *P value < 0.05. (**B**–**E**) Scatterplots of significant Spearman’s rank correlation of serum anti-ADAMTSL5 autoantibody concentration with Foxp3 expression by CD3 + CD4 + CD25 + CD127^lo^ T cells derived from peripheral blood of psoriasis (n = 21) and PsA patients (n = 20). Differentiation between Foxp3^int^ (**B**, **C**) and Foxp3^hi^ (**D**, **E**) Tregs in patients with psoriatic disease (**B**, **D**) versus PsA patients only (**C**, **E**). ADAMTSL5: A Disintegrin And Metalloprotease domain containing ThromboSpondin type 1 motif-Like 5; Foxp3^int^/^-hi^: forkhead box P3 expression intermediate / high; HC: healthy control; IgG: Immunoglobulin G; P: p value; PsA: psoriatic arthritis; PsA: psoriatic arthritis; PsO: psoriasis; ρ: Spearman’s rho.

Lastly, we queried whether Treg phenotypical variances are associated with loss of peripheral tolerance in PsA. To investigate this further, we quantified autoantibodies in serum and synovial fluid against a newly discovered autoantigen specific for psoriatic disease: A Disintegrin And Metalloprotease domain containing ThromboSpondin type 1 motif-Like 5 (ADAMTSL5)(patient characteristics shown in Supplemental Table S3 and S4)^33^. ADAMTSL5 is a protein present in extracellular matrix and implicated in microfibril function modulation^34^. We observed higher anti-ADAMTSL5 IgG in PsA serum (575 µg/mL (IQR 321–1523)), as compared to HC serum (205 µg/mL (IQR 28–833), P = 0.004), psoriasis serum (319 µg/mL (IQR 103–645), P = 0.012) and PsA synovial fluid (138 µg/mL (IQR 77–348), *P* =  < 0.0001)(Fig. 6A, Supplemental Figure S6A). ADAMTSL5 autoantibodies discriminated between psoriasis and PsA diagnosis with an AUROC of 0.67 (95%CI 0.543–0.787), P = 0.012) (Supplemental Figure S6B). No associations of anti-ADAMTSL5 IgG with clinical characteristics or disease activity were observed (*data not shown*).

Further, we identified an association of Foxp3 instability with autoantibody production in psoriatic disease. We observed that the Foxp3^int^ Treg subset correlated with the presence of ADAMTSL5 autoantibodies in peripheral blood of patients with psoriatic disease (Spearman’s rho (ρ) 0.466, P = 0.002)(Fig. 6B). The correlation was even stronger in PsA alone (ρ 0.606, P = 0.005)(Fig. 6C). In contrast, we observed an inverse correlation of Foxp3^hi^ Tregs with anti-ADAMTSL5 IgG in psoriatic disease (ρ -0.380, P = 0.014)(Fig. 6D), again more pronounced in PsA alone (ρ -0.528, P = 0.017)(Fig. 6E). Absence of a correlation in healthy controls suggests that the association of Foxp3 expression by Tregs with ADAMTSL5 autoantibodies is specific for psoriatic disease (Supplemental Figure S7).

## Discussion

In the inflammatory microenvironment of autoimmune disease, Treg defects and differentiation are suggested to play a role in loss of peripheral immune tolerance. However, the implications of Treg dysfunction and plasticity have not been clarified in psoriatic disease. To our knowledge, this is the first study to perform in-depth phenotypical characterization of Tregs derived from the inflammatory microenvironment of inflamed joints in patients with psoriatic disease. Here, we provide evidence for Treg variance in PsA by showing distinct phenotypical and functional properties of intra-articular Tregs as compared to Tregs in circulation: downregulation of key transcription factor Foxp3, increased cytokine production, upregulation of inhibitory immune receptors, and upregulation of markers that have been reported to associate with a pro-inflammatory potential of Tregs: CD161, RORγt and ICOS^7,14,15,30,31,37–39^.

Foxp3 is the key transcription factor of Tregs and its expression is essential for Treg development, maintenance and function^29^. Our results demonstrate a significant increase of intra-articular Tregs with intermediate Foxp3 expression in PsA patients. Association of Foxp3 with PsA has been previously described by one study, that identified a hemizygous Foxp3 mutation (c.1222G > A) in familial juvenile PsA^35^. Moreover, in psoriasis patients, it was shown that enhanced loss of Foxp3 is linked to Treg differentiation into IL-17A producing cells^15^. In the broader context of autoimmune disease, multiple studies suggested that stability of Foxp3 expression is negatively affected by pro-inflammatory conditions^13,19^. These findings have clinical relevance, because Treg defects—including Foxp3 instability—could contribute to disease pathophysiology. This contribution is either through increased escape of autoreactive T cells from Treg regulation or, what has been suggested more recently, by conversion of Tregs into autoreactive, memory T cells^13,15,29^.

Furthermore, we show that the intra-articular subset of Tregs with lower Foxp3, as compared to their Foxp3^hi^ counterparts in the same tissue location, have lower expression of the inhibitory receptors CTLA-4 and TIGIT. These Foxp3^int^ Tregs produce even more of the psoriatic disease hallmark cytokine IL-17, and display the highest expression of CD161. These substantial differences between Foxp^int^ and Foxp3^hi^ are relevant, since our results demonstrate that Tregs with decreased Foxp3 expression are present in large numbers in the synovial compartment. In line with the homeostatic importance of Tregs in psoriatic disease, we observed a relation of Foxp3 instability with loss of immune tolerance in psoriatic disease: presence of ADAMTSL5 antibodies in psoriasis and PsA reflected the balance between Foxp^int^ and Foxp3^hi^ Tregs. However, as we did not perform functional experiments and only studied relative Foxp3 expression, we are careful to draw definite conclusions.

Based on their phenotypical characteristics, synovial fluid-derived Tregs—and in particular the Foxp3^int^ Treg subset—might play a role in pathogenesis or ongoing inflammation in PsA. First, because failure to upregulate inhibitory receptors has been shown to contribute to the comprised suppressive function of intra-articular Tregs in inflammatory arthritis^8^. In addition, CD161 + Tregs were previously identified as a subset capable of IL-17A and IFNγ production, and to exhibit a pro-inflammatory potential^7,30^. In fact, CD161 + Tregs are the predominant IL-17 producing Treg population in inflamed joints of inflammatory arthritis patients^7^. Furthermore, concerning the implications of RORγt + Tregs, in inflammatory bowel disease it was shown that the capacity of IL-17 + RORγt + Tregs to suppress autologous T cell proliferation is reduced by approximately 60%^14^. Also, RORγt expression associates with IL-17A producing Tregs in psoriasis^15^. These findings are not surprising, considering that Foxp3 and RORγt transcription factors drive differentiation of T cells towards Tregs or Th17 cells^36^. With regards to functional differences, intra-articular Tregs may contribute to ongoing localized inflammation by increased production of IL-17A, as compared to peripheral Tregs. IL-17 has previously been associated with unresponsiveness of Teff in the microenvironment of inflammatory arthritis^18^. Taken together, we identified a distinct phenotype of synovial fluid-derived Tregs, most pronounced in the Treg subset with downregulated Foxp3. The phenotypical characteristics of these Tregs warrant further investigation to elucidate their role in PsA pathogenesis.

Further, the association of ICOS + Tregs with PsA disease activity drew our attention, because ICOS is most commonly associated with a strongly inhibitory Treg subset^32^. However, studies in the last decade have suggested a possible role for ICOS + Tregs in pathogenesis, contributing to autoimmune rheumatic disease. In lupus, RA and spondyloarthritis, associations were found of ICOS expression by Tregs with high disease activity, with non-response to therapy, increased autoantibodies, and pro-inflammatory cytokine production^31,37–39^. Since we show that all intra-articular Tregs upregulate ICOS expression, even independent of Foxp3 expression, further investigation is warranted for ICOS + Tregs as possible therapeutic target for treatment of psoriatic disease.

Our study has several limitations. First, we were limited by the number of subjects tested, especially concerning synovial fluid mononuclear cell samples, and future studies to confirm our results are warranted. However, we deem that the evident observed dissimilarities between Tregs derived from synovial fluid and peripheral blood have enabled us to draw conclusions about Treg variances. Second, the use of DMARDs (methotrexate and golimumab) by two patients in our SFMC cohort could have influenced our results, although contradicting results have been published as to whether DMARDs affect Treg phenotype and function^40–42^. Third, we have not assessed the pro-inflammatory potential of Tregs in functional experiments or performed assays to evaluate the suppressive capacity of intra-articular Tregs. Fourth, with flow cytometry analyses we could only assess relative differences. Hence, we can only speculate about the implications of absolute numbers of this distinct subset of Tregs in PsA pathogenesis. Fifth, we observed markedly lower Treg CD161 expression as compared to literature, which may be the result of our gating strategy that selected only Foxp3 + CD25 + CD127^lo^ T cells in an attempt to exclude activated non-Treg T cells with low Foxp3 expression. Moreover, this gating strategy might have resulted in the inclusion of a minor subset of effector T cells, that upon activation might transiently express CD25 and Foxp3^43^. Nevertheless, based on what is known from literature in other types of inflammatory arthritis, we deem to have identified an interesting Treg subset that warrants further investigation.

Since we have not confirmed our hypothesis by assessing the suppressive capacity of intra-articular Tregs, it must be taken into consideration that even differentiated Tregs may be able to effectively suppress Teff^6,7,16^. If and how differentiated Tregs in inflammatory arthritis can effectively suppress Teff T cells is an increasing topic of interest and contradicting results have been published^44^. Some concluded that impaired expression of immune regulatory molecules or lack of cytokine production are key to defective Teff suppression by Tregs^8,9,45^. Others attributed failure of effective Teff suppression to the factor that Tregs are prone to apoptosis under inflammatory conditions^44^. Moreover, evidence suggested that sustained resistance of local CD4 + and CD8 + Teff in an inflammatory microenvironment could be key to ineffective Treg suppression^18^. Whether these mechanisms play a role in psoriatic disease has yet to be elucidated. In the case that an important role for Tregs derived from sites of inflammation in PsA pathogenesis is confirmed, this may facilitate identification of new treatment targets and therapies, including Treg growth factors, Treg stabilizing factors and therapies that enhance Treg function^46^. In preclinical models and clinical trials low dose IL-2 and cellular therapy with polyclonal, therapeutic Tregs have already shown promising results^46–48^. Moreover, research to artificially stabilize Treg Foxp3 expression in vitro for clinical applications are ongoing^49^. As treatment options for autoimmune disease are evolving, we deem it essential to further advance our understanding of the role of Tregs in psoriatic disease pathogenesis.

## Conclusions

In conclusion, we show that Tregs derived from the inflammatory environment of inflamed joints in PsA patients exhibit a distinct phenotype characterized by increased expression of CD161, RORγt and ICOS. Moreover, we identify the importance of Foxp3 expression by Tregs, with a novel role for Foxp3^int^ Tregs with a heightened capacity to produce IL-17A.

## Supplementary Information

Supplementary Information.

## Data Availability

All data generated or analyzed during this study are included in this published article and its supplementary information files.

## Abbreviations

ADAMTSL5: A disintegrin and metalloprotease domain containing thrombospondin type 1 motif-like 5
CTLA-4: Cytotoxic T lymphocyte-associated protein 4 (CD152)
DMARD: Disease modifying anti-rheumatic drug
ELISA: Enzyme-linked immuno sorbent assay
Foxp3: Forkhead box p3
ICOS: Inducible T cell costimulator (CD278)
IL: Interleukin
OA: Osteoarthritis
PASDAS: Psoriatic arthritis disease activity score
PsA: Psoriatic arthritis
RA: Rheumatoid arthritis
RORγt: Retinoic acid–related orphan receptor γ
Teff: Effector T cell
TIGIT: T cell immunoreceptor with Ig and ITIM domains
Tregs: Regulatory T cells
