# Author Correction: Regulatory T cells in psoriatic arthritis: an IL-17A-producing, Foxp3^int^CD161 + RORγt + ICOS + phenotype, that associates with the presence of ADAMTSL5 autoantibodies

**DOI:** 10.1038/s41598-023-28623-y

**Published:** 2023-01-24

**Authors:** Juliëtte N. Pouw, Michel A. M. Olde Nordkamp, Tessa van Kempen, Arno N. Concepcion, Jacob M. van Laar, Femke van Wijk, Julia Spierings, Emmerik F. A. Leijten, Marianne Boes

**Affiliations:** 1 Department of Rheumatology and Clinical Immunology, University Medical Center Utrecht, Utrecht University, H03.103, P.O. Box 85500, 3508 GA Utrecht, The Netherlands; 2 Center for Translational Immunology, University Medical Center Utrecht, Utrecht University, 3508 AB Utrecht, The Netherlands; 3 Biomedical Laboratory Sciences, Avans University of Applied Sciences, 4800 RA Breda, The Netherlands; 4 Department of Rheumatology, Sint Maartenskliniek, 6500 GM Nijmegen, The Netherlands; 5 Department of Pediatric Immunology, Wilhelmina Children’s Hospital, Utrecht University, 3508 AB Utrecht, The Netherlands

Correction to: *Scientific Reports* 10.1038/s41598-022-24924-w, published online 30 November 2022

The original version of this Article contained errors in the spelling of the authors Juliëtte N. Pouw, Michel A. M. Olde Nordkamp, Tessa van Kempen, Arno N. Concepcion, Jacob M. van Laar, Femke van Wijk, Julia Spierings, Emmerik F. A. Leijten & Marianne Boes, which were incorrectly given as J. N. Juliëtte Pouw, M. A. M. Michel Olde Nordkamp, T. Tessa van Kempen, A. N. Arno Concepcion, J. M. Jacob van Laar, F. Femke van Wijk, J. Julia Spierings, E. F. A. Emmerik Leijten & M. Marianne Boes respectively.

The original Article has been corrected.

