# Supplementary material for: Regulatory T cells in psoriatic arthritis: an IL-17A-producing, Foxp3intCD161 + RORγt + ICOS + phenotype, that associates with the presence of ADAMTSL5 autoantibodies
Source: Sci Rep. 2022 Nov 30;12:20675. doi: 10.1038/s41598-022-24924-w (PMC9712434; doi:10.1038/s41598-022-24924-w)
Supplement: Supplementary file 1 — Supplementary Information. [file 41598_2022_24924_MOESM1_ESM.docx]

## SUPPLEMENTAL DATA

**Supplemental Figure S1.** *FMO controls of Foxp3, Ki67, CTLA-4, TIGIT and ICOS as measured by flow cytometry*


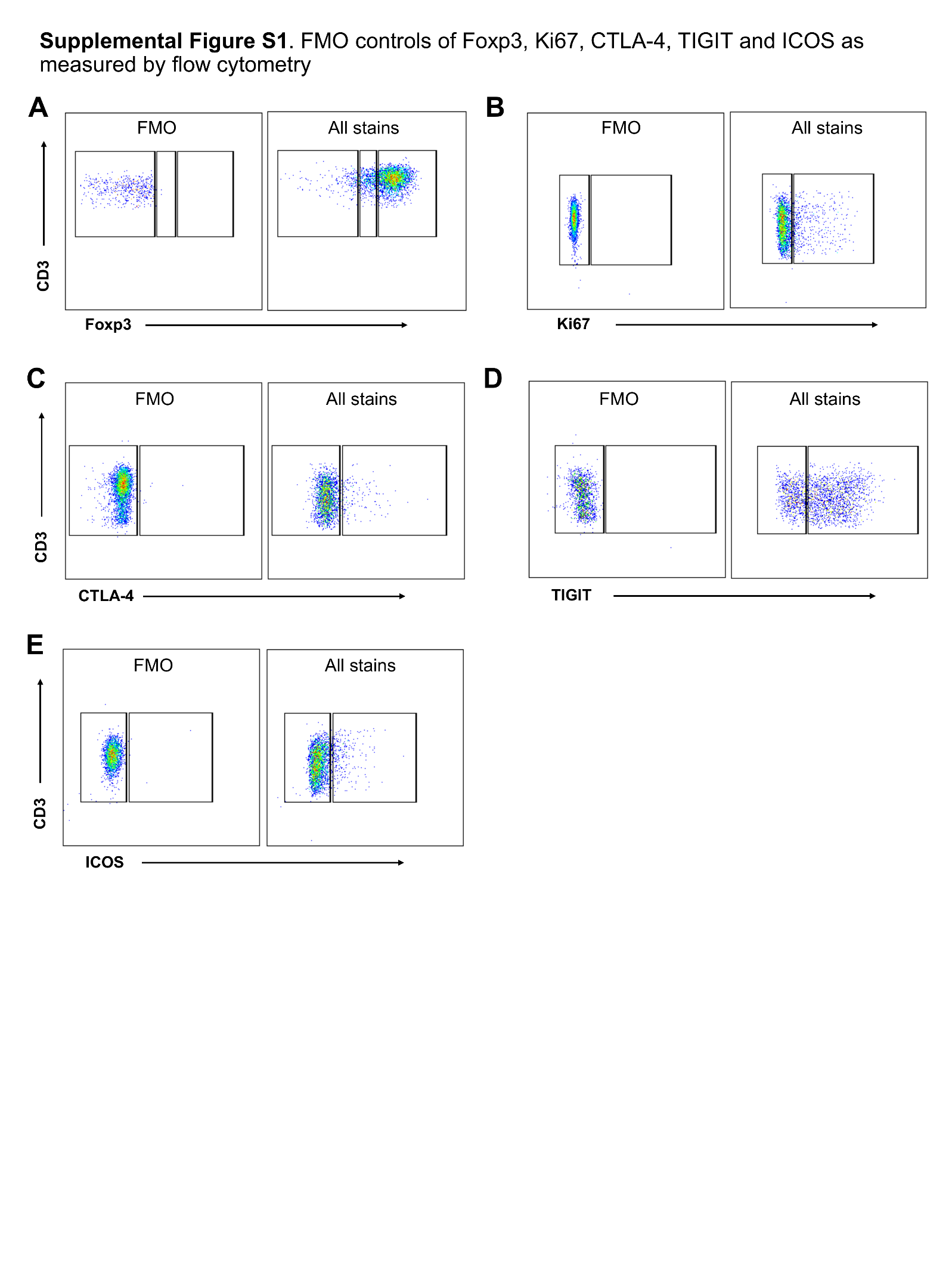
***Legend****: Representative flow cytometry dot plots of CD4+CD25+CD127^lo^ T cells derived from peripheral blood of healthy controls and psoriatic arthritis patients. FMO controls of (****A****) Foxp3, (****B****) Ki67, (****C****) CTLA-4, (****D****) TIGIT and (****E****) ICOS.* ***Abbreviations****: CTLA-4: cytotoxic T-lymphocyte-associated protein 4 (CD152); FMO: Fluorescence minus one; Foxp3: forkhead box P3; ICOS: inducible T-cell costimulator (CD278); Ki67: antigen KI67; T cell immunoreceptor with Ig and ITIM domains.*

**Supplemental Figure S2.** *FMO, healthy and medium controls of CD161, RORγt, IL-10 and IL-17A as measured by flow cytometry*


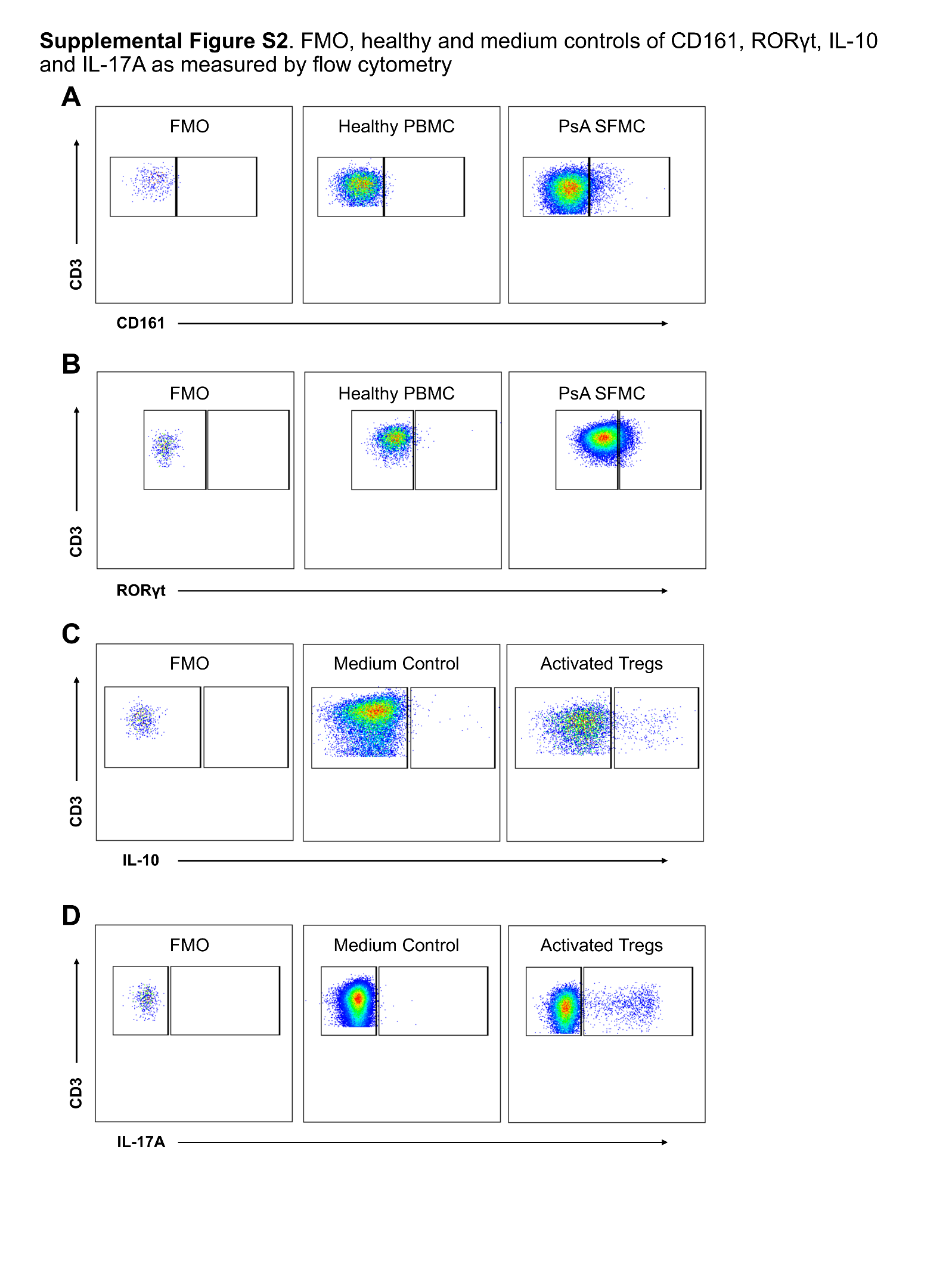


***Legend****: Representative flow cytometry dot plots of CD4+CD25+CD127^lo^ T cells derived from peripheral blood of healthy controls and psoriatic arthritis patients. (****A,B****) Comparison CD161 (A) and RORγt (B) expression between FMO controls, representative healthy controls and psoriatic arthritis samples. (****C,D****) Comparison IL-10 (C) and IL-17A (D) expression between FMO controls, medium controls and PMA/ionomycin stimulated Tregs.* ***Abbreviations****: Fluorescence minus one; IL: interleukin; Ki67: antigen KI67; RORγt: retinoic acid receptor-related orphan receptor gamma; Treg: T regulatory cell.*

**Supplemental Figure S3.** *Correlation of Foxp3^int^ with Foxp3^hi^ Tregs in peripheral blood and synovial fluid of HC, PsO and PsA patients*


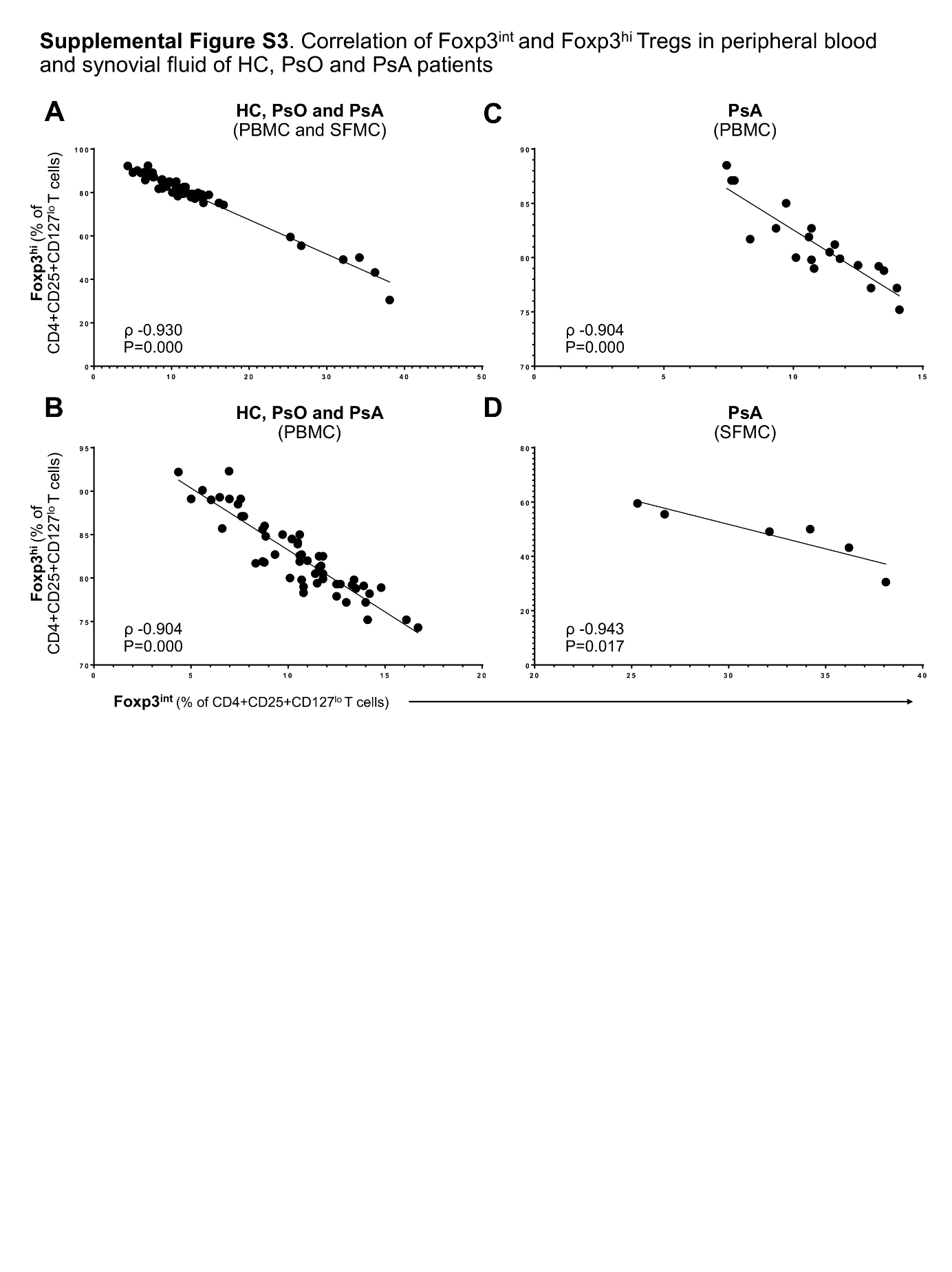


***Legend****: Flow cytometry analysis of Foxp3 expression by CD4+CD25+CD127^lo^ T cells derived from peripheral blood of HC (n=13), psoriasis patients (n=21) and PsA patients (n=20), and from synovial fluid of PsA patients (n=6). Shown are scatter plots of the proportion of Tregs with Foxp3^int^ and Foxp3^hi^ expression. Correlation was tested using Spearman’s rho (ρ). Included samples are (****A****) pooled PBMC and SFMC of HC, PsO and PsA patients, (****B****) pooled PBMC of HC, PsO and PsA patients, (****C****) PBMC of PsA patients and (****D****) SFMC of PsA patients.* ***Abbreviations****: Foxp3^int^ / ^-hi^: forkhead box P3 expression intermediate / high; HC: healthy control; PBMC: peripheral blood mononuclear cells; PsA: psoriatic arthritis; PsO: psoriasis; SFMC: synovial fluid mononuclear cells.*

**Supplemental Figure S4.** *Increase of proliferating Tregs in PsA synovial fluid, as compared to circulation*


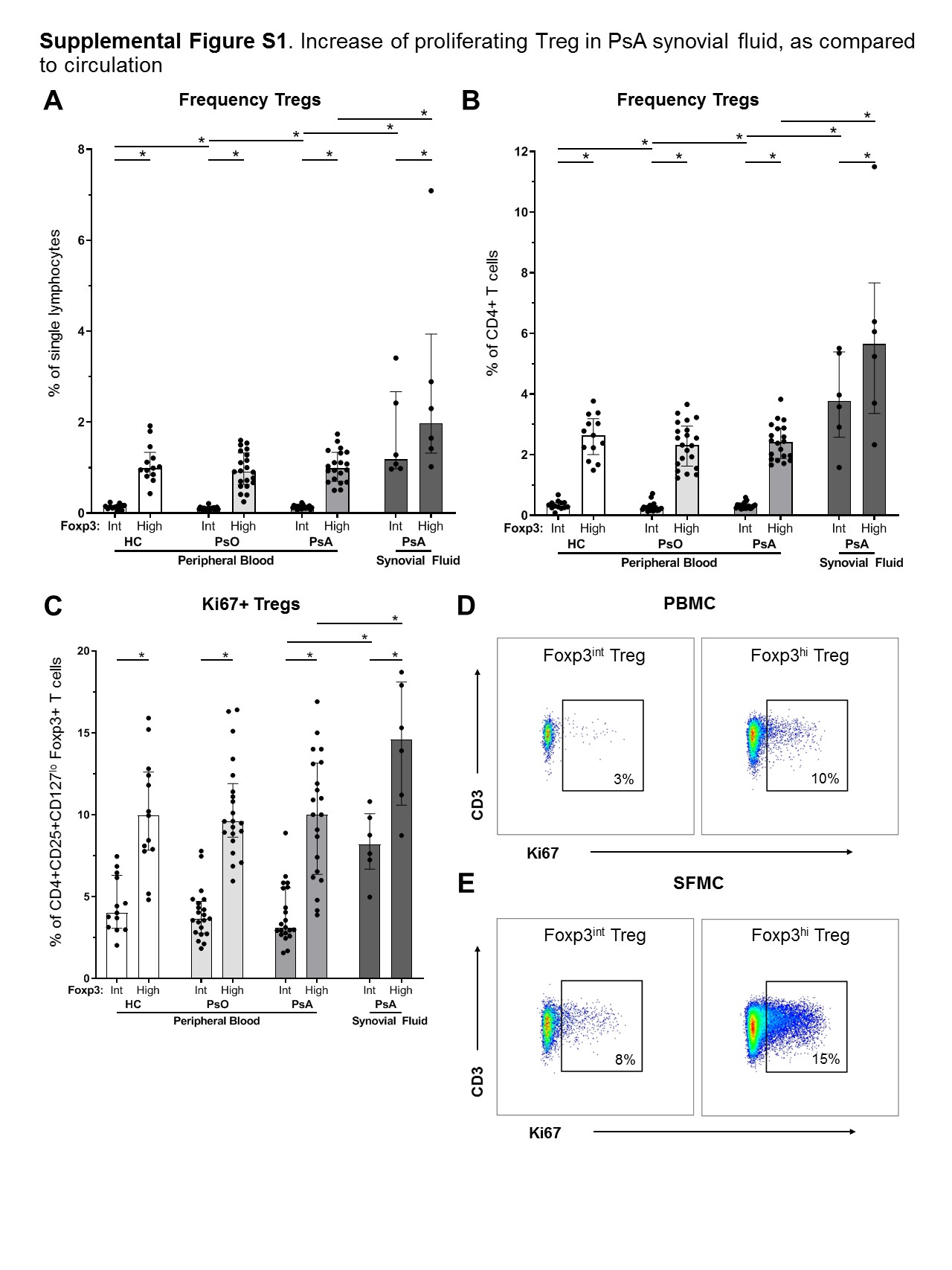


***Legend****:* *Flow cytometry analysis of CD4+CD25+CD127^lo^Foxp3+ Tregs derived from peripheral blood of HC (n=13), psoriasis patients (n=21) and PsA patients (n=21), and from synovial fluid of PsA patients (n=6). Bar graphs: symbols represent individual subjects; bars show median with interquartile range; * P value <0.05 (PsA synovial fluid only compared with PsA peripheral blood). (****A,B****) Proportion of Foxp3^int^ and Foxp3^hi^ Tregs of single lymphocytes (A) and CD4+ T cells (B). (****C****) Proportions of Ki67+Foxp3^int^ and Ki67+Foxp3^hi^ Tregs. (****D,E****) Representative flow cytometry plots to identify Ki67+Foxp3^int^ and -Foxp3^hi^ Tregs derived from peripheral blood (D) and synovial fluid (E). Percentages in PBMC dot plots represent median of PB-derived Tregs in PsA. Percentages in SFMC dot plots represent median of SF-derived Tregs.* ***Abbreviations****:* *Foxp3^int^ / ^hi^: forkhead box P3 expression intermediate / high; HC: healthy control; Int: intermediate expression of Foxp3; Ki67: antigen KI67; MFI: median fluorescent intensity; PBMC: peripheral blood mononuclear cells; PsA: psoriatic arthritis; PsO: psoriasis; SFMC: synovial fluid mononuclear cells; Tregs: T regulatory cells.*

**Supplemental Figure S5.** *Treg cytokine production*


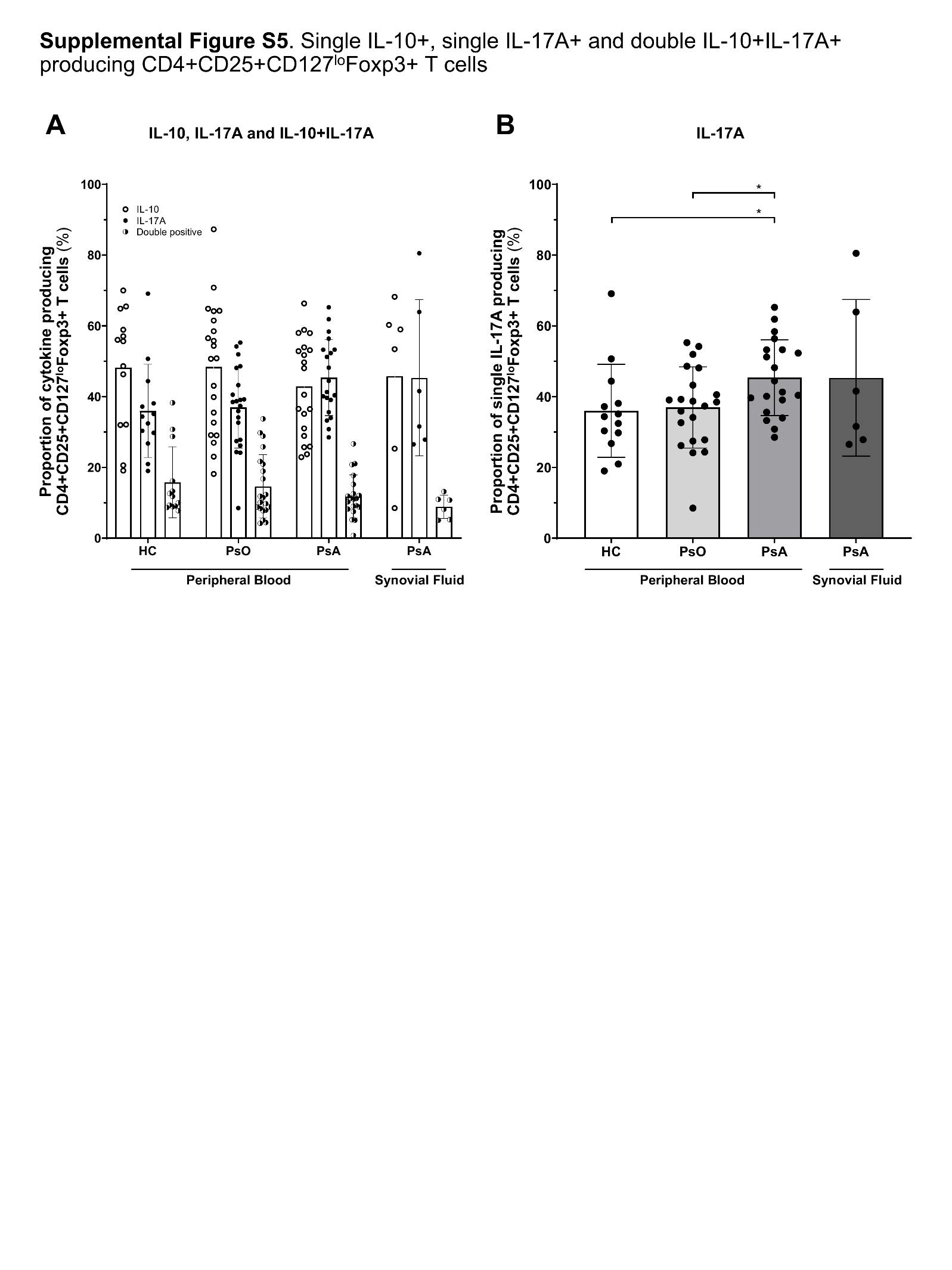
***Legend****: Flow cytometry analysis of cytokine-producing CD4+CD25+CD127^lo^Foxp3+ Tregs derived from peripheral blood of HC (n=13), psoriasis patients (n=21) and PsA patients (n=20), and from synovial fluid of PsA patients (n=6). PBMC and SFMC were cultured for 4,5 hours with 20 ng/mL PMA, 1 µg/ml ionomycin and 1:1000 BD GolgiStop. Symbols represent individual subjects; bars show median with interquartile range; * P value <0.05 (synovial fluid only compared with PsA peripheral blood). (****A****) Proportion of CD4+CD25+CD127^lo^Foxp3+ Tregs, that upon activation produce IL-10, IL-17A or both. (****B****) Proportion of CD4+CD25+CD127^lo^Foxp3+ Tregs, that upon activation produce IL-17A.* ***Abbreviations****: HC: healthy control; IL: interleukin; PsA: psoriatic arthritis; PsO: psoriasis; SFMC: synovial fluid mononuclear cells; Tregs: T regulatory cells.*

**Supplemental Figure S6.**
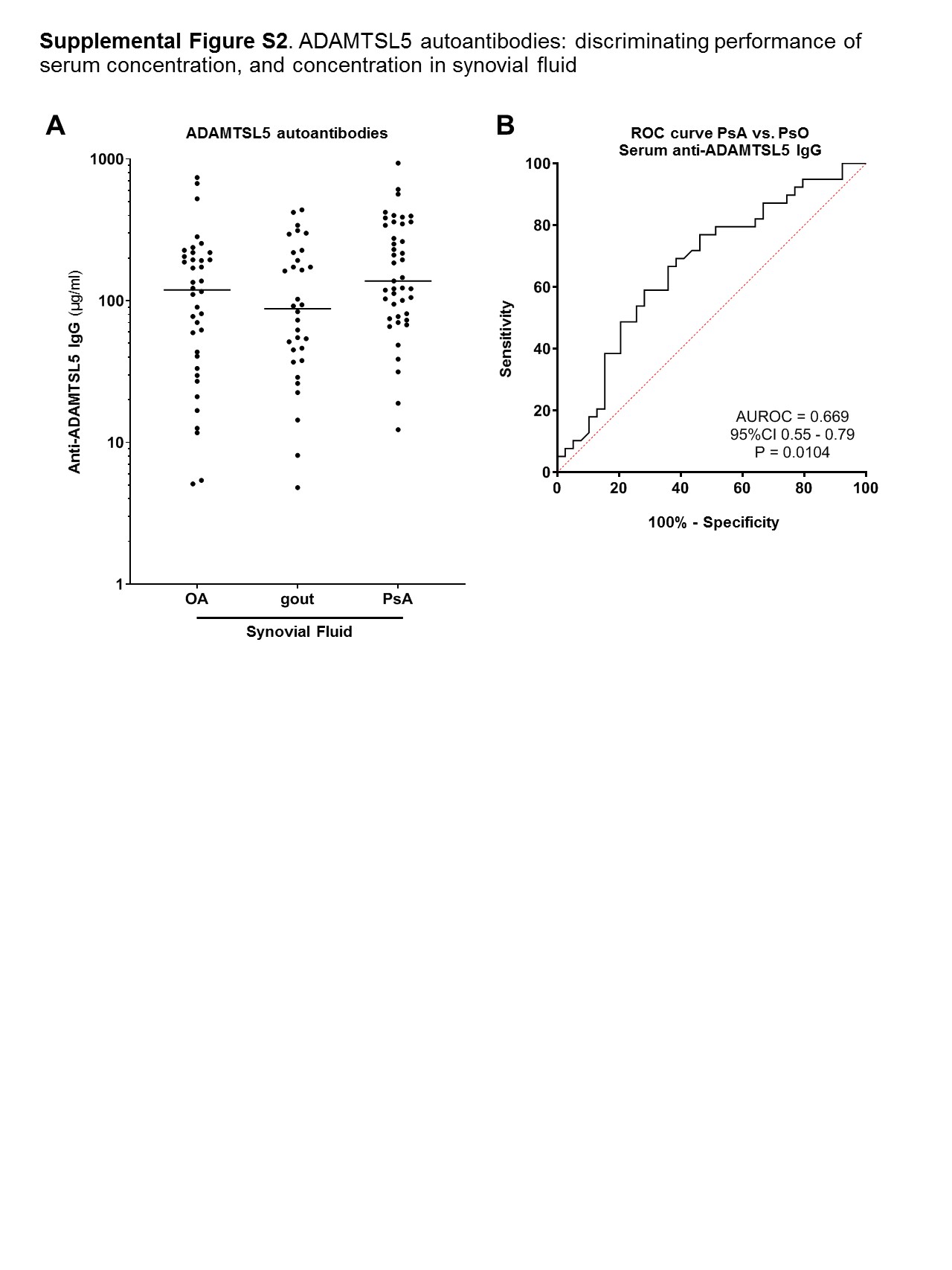
 *ADAMTSL5 autoantibodies: quantification in synovial fluid and discriminating performance of serum concentration*

***Legend****: ELISA of anti-ADAMTSL5 IgG. (****A****) Autoantibody concentration in synovial fluid of OA (n=38), gout (n=32) and PsA patients (n=43). (****B****) Discriminating performance between PsA and psoriasis of serum IgG autoantibodies against ADAMTSL5 in patients with psoriatic disease (psoriasis n=39, PsA n=39).* ***Abbreviations****: ADAMTSL5: A Disintegrin And Metalloprotease domain containing ThromboSpondin type 1 motif-Like 5; AUROC: area under the receiver operating characteristic curve; CI: confidence interval; IgG: Immunoglobulin G; OA: osteoarthritis; P: p value; PsA: psoriatic arthritis; PsO: psoriasis.*

**Supplemental Figure S7.** *No association of Foxp3 downregulation by Tregs with ADAMTSL5 autoantibodies in healthy controls*


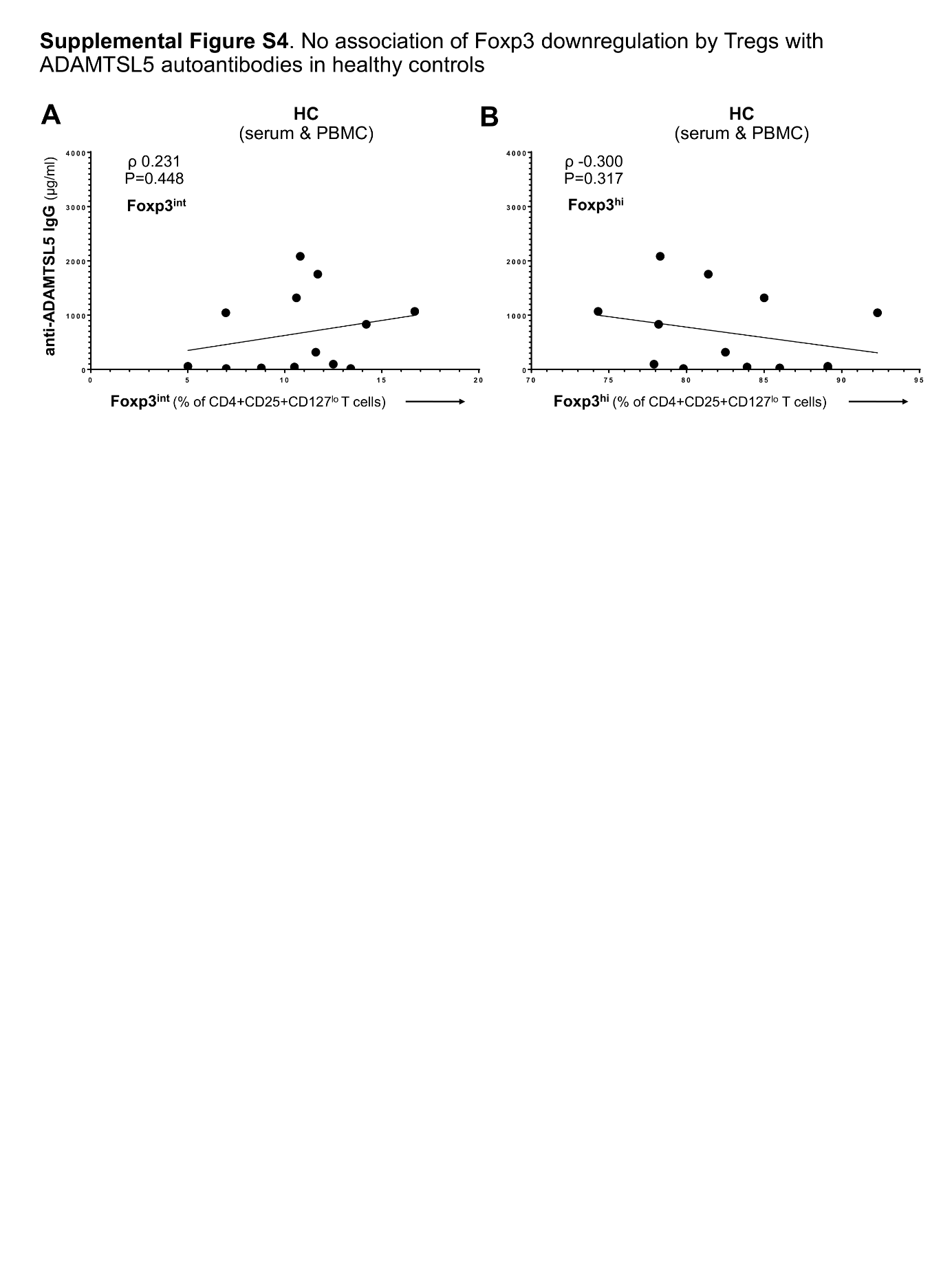


**Legend**: Association of ADAMTSL5 autoantibodies (as measured by ELISA) with Foxp3 expression by *Tregs (as measured by flow cytometry) in healthy controls (n=13). Shown are scatterplots of serum anti-ADAMTSL5 IgG autoantibody concentration (µg/mL) with the proportion of Tregs that express intermediate Foxp3 (****A****) and the proportion of Tregs that express high Foxp3 (****B****).* ***Abbreviations****: ADAMTSL5: A Disintegrin And Metalloprotease domain containing ThromboSpondin type 1 motif-Like 5; Foxp3^int^ / ^-hi^: forkhead box P3 expression intermediate / high; HC: healthy control; IgG: Immunoglobulin G; P: p value; PBMC: peripheral blood mononuclear cells; PsA: psoriatic arthritis; PsO: psoriasis; ρ: Spearman’s rho.*

**Supplemental Table S1.** *Antibodies flow cytometry*

| **Target** (label, category number, company) | **Dilution** (1:x) | **Stain** |
| --- | --- | --- |
| **Panel I** |  |  |
| CD127 (BV605; 2356670; Sony Biotechnology) | 20 | Cell surface |
| CD152 (PE; 555853; BD) | 12.5 | Cell surface |
| CD25 (PE-Cy7; 557741; BD) | 25 | Cell surface |
| CD278 (APC; 17-9948-42; eBioscience) | 20 | Cell surface |
| CD3 (AF700; 300424; Biolegend) | 50 | Cell surface |
| CD4 (BV785; 300554; Biolegend) | 50 | Cell surface |
| Fixable Viability Dye (eF506; 65-0866-14; eBioscience) | 1000 | Cell surface |
| Foxp3 (eF450; 48-4776-42; eBioscience) | 50 | Intra-cellular |
| Ki67 (FITC; F7268; DAKO) | 50 | Intra-cellular |
| TIGIT (PerCP-eF710; 46-9500-42; eBioscience) | 50 | Cell surface |
| **Panel II** |  |  |
| CD127 (BV421; 562436; BD) | 40 | Cell surface |
| CD161 (BV510; 563212; BD) | 50 | Cell surface |
| CD25 (BV711; 563159; BD) | 50 | Cell surface |
| CD4 (PerCP; 300528; Biolegend) | 20 | Intra-cellular |
| Fixable Viability Dye (eF780; 65-0865-14, eBioscience) | 1000 | Cell surface |
| Foxp3 (PE-CF594; 562421; BD) | 20 | Intra-cellular |
| IL-10 (PE; 554706; BD) | 20 | Intra-cellular |
| IL-17A (FITC; 11-7179-82; eBioscience) | 40 | Intra-cellular |
| RORγt (APC; 17-6988-82; eBioscience) | 60 | Intra-cellular |
|  |  |  |

**Supplemental Table S2.** *Baseline characteristics flow cytometry cohort*

| **Characteristic** | **Peripheral blood** | | | **Synovial Fluid** |
| --- | --- | --- | --- | --- |
|  | **HC**  (n=13) | **PsO**  (n=21) | **PsA**  (n=21) | **PsA**  (n=6) |
| Age (y) * | 46 ± 15 | 39 ± 14 | 49 ± 11 | 50 ± 10 |
| Female, n | 9 (69%) | 10 (48%) | 7 (33%) | 0 (0%) |
| DMARD use, n ** | - | 0 (0%) | 0 (0%) | 2 (33%) ^a)^ |
| PsO duration (y) * | - | 5.5 (2.3-20.2) | 21.9 (9.5-31.4) | 24.0 (1.6-51.8) |
| PsA duration (y) | - | - | 3.0 (0.1-8.6) | 6.8 (4.7-12.2) |
| BMI (kg/m^2^) | - | 25.7 ± 2.8 | 26.3 (24.6-30.7) | 27.9 (23.5-33.7) |
| CRP (mg/L) | - | 1.6 (1.1-3.7) | 2.1 (1.4-3.5) | 3 (1.3-.) |
| ESR (mm/h) | - | 5 (2-8) | 8 (5-13) | 8 (5-15) |
| ACPA positive, n | - | 0 (0%) | 0 (0%) | 0 (0%) |
| RF positive, n | - | 0 (0%) | 1 (7%) | 0 (0%) |
| PASI * | - | 5.2 (1.6-8.9) | 1.9 (0.9-3.0) | 3.7 ^d)^ |
| Tender joints (78) * | - | 0 (0-0) | 4 (1-9) | 1 (1-1) |
| Swollen joints (76) * | - | 0 (0-0) | 3 (1-7) | 1 (1-2) |
| Nail psoriasis, n | - | 10 (48%) | 13 (65%) | 3 (50%) |
| Dactylitis, n | - | 0 (0%) | 2 (11%) | 0 (0-0) |
| Dactylitis ever, n * | - | 0 (0%) | 6 (32%) | 1 (17%) |
| LEI | - | 0 (0-0) | 0 (0-0) | 0 (0-0) |
| Enthesitis ever, n | - | 5 (24%) | 5 (40%) | 3 (60%) |
| Erosions, n * | - | 0 (0%) | 8 (42%) | 2 (40%) |
| HAQ | - | 0.0 (0.0-0.75) | 0.38 (0.13-1.00) | 0.25 ^d)^ |
| DLQI | - | 7.5 (3.5-13.3) | 3.0 (1.0-5.0) | 2.0 ^d)^ |
| SF-36 PCS | - | 54 (44-58) | 48 (45-53) | 33.2 ^d)^ |
| SF-36 MCS | - | 53 (38-56) | 54 (46-58) | 61.8 ^d)^ |
| DAPSA ^b)^ | - | - | 14.3 (12.1-27.1) | 16.3 ^d)^ |
| PASDAS ^c)^ | - | - | 5.5 (3.3-4.8) | 5.4 ^d)^ |

***Legend****: Presented data are from time of visit, unless otherwise indicated. Categorical data are presented with frequencies (%) and continuous data are presented as mean ± SD (normally distributed variables) or median (IQR)(non-normally distributed variables). * P value PsO vs. PsA <0.05. ** P value PsA PB vs. SF <0.05.* ***Footnotes****: ^a^) Golimumab (n=1) and methotrexate (n=1). ^b)^ DAPSA: (TJC68 + SJC66 + VAS PGA (range 0-10) + VAS pain (range 0-10) + CRP (range 0-10 mg/dL))). ^c)^ PASDAS: (((0.18 √(VAS PhGA (range 0-100)) + (0.159 √(VAS PGA (range 0-100)) – (0.253 * √(SF-36 PCS)) + (0.101 * ln(SJC66 +1)) + (0.048 * ln(TJC68 +1)) + (0.23 * ln(enthesitis count+1)) + (0.37 * ln(dactylitis count+1)) + (0.102 * ln(CRP (mg/L) +1)) +2) * 1.5). ^d)^ Data available from n=1.* ***Abbreviations****: ACPA: anti-citrullinated protein antibodies; BMI: body mass index; CRP: C-reactive protein; DAPSA: disease activity index for PsA (range 0-164); DMARD: disease modifying anti-rheumatic drug use past three months; Erosions: reported by radiologist on conventional radiography and/or magnetic resonance imaging; ESR: erythrocyte sedimentation rate; HAQ: health assessment questionnaire (range 0-3); HC: healthy control; LEI: leeds enthesitis index (range 0-6); PASDAS: psoriatic arthritis disease activity score (range 0-10); PASI: psoriasis severity index (range 0-72); PsA: psoriatic arthritis; PsO: psoriasis; RF: rheumatoid factor.*

**Supplemental Table S3.** *Baseline characteristics ELISA cohort – serum*

| **Characteristic** | **HC**  (n=35) | **PsO**  (n=39) | **PsA**  (n=39) |
| --- | --- | --- | --- |
| Age (years) | 44 ± 13 | 42 ± 16 | 44 ± 13 |
| Female, n (%) * | 17 (50%) | 22 (56%) | 12 (31%) |
| DMARD use, n (%) | 0 (0%) | 0 (0%) | 0 (0%) |
| PsO duration (y) | - | 10.2 (3.7-21.1) | 13.8 (7.0-25.8) |
| PsA duration (y) | - | - | 1.0 (0.1-5.4) |
| BMI (kg/m^2^) | - | 29.2 ± 7.2 | 27.5 ± 3.6 |
| CRP (mg/L) | - | 2.1 (1.0-5.4) | 3.0 (1.9-4.6) |
| ESR (mm/hour) | - | 6 (2-11) | 8 (3-13) |
| ACPA positive, n | - | 0 (0%) | 1 (4%) |
| RF positive, n | - | 1 (14%) | 2 (7%) |
| PASI | - | 3.9 (1.7-8.2) | 2.5 (1.0-4.2) |
| Tender joints (78) | - | 0 (0-1) | 3 (0-7) |
| Swollen joints (76) | - | 0 (0-0) | 2 (0-7) |
| Nail psoriasis, n |  | 23 (59%) | 24 (65%) |
| Dactylitis, n * | - | 0 (0%) | 6 (17%) |
| Dactylitis ever, n | - | 1 (3%) | 13 (36%) |
| LEI * ^a)^ | - | 0 (0-0) | 0 (0-0) |
| Enthesitis ever, n | - | 8 (21%) | 22 (58%) |
| Erosions, n | - | 0 (0%) | 12 (32%) |
| HAQ | - | 0.0 (0.0-0.7) | 0.5 (0.3-1.0) |
| DLQI | - | 8 (2-12) | 3 (1-7) |
| SF-36 PCS | - | 53.0 (41.3-57.2) | 47.2 (38.3-51.5) |
| SF-36 MCS | - | 49.9 (36.7-56.6) | 55.0 (48.0-58.7) |
| DAPSA ^b)^ | - | - | 14.3 (11.0-26.9) |
| PASDAS ^c)^ | - | - | 4.6 (3.6-5.3) |

***Legend****: Presented data are from time of visit, unless otherwise indicated. Categorical data are presented with frequencies (%) and continuous data are presented as mean ± SD (normally distributed variables) or median (IQR)(non-normally distributed variables). * P value PsO vs. PsA <0.05.* ***Footnotes****: ^a^) LEI mean: PsO 0.1 ± 0.3; LEI mean PsA 0.4 ± 0.8. ^b)^ DAPSA: (TJC68 + SJC66 + VAS PGA (range 0-10) + VAS pain (range 0-10) + CRP (range 0-10 mg/dL))). ^c)^ PASDAS: (((0.18 √(VAS PhGA (range 0-100)) + (0.159 √(VAS PGA (range 0-100)) – (0.253 * √(SF-36 PCS)) + (0.101 * ln(SJC66 +1)) + (0.048 * ln(TJC68 +1)) + (0.23 * ln(enthesitis count+1)) + (0.37 * ln(dactylitis count+1)) + (0.102 * ln(CRP (mg/L) +1)) +2) * 1.5).* ***Abbreviations****: ACPA: anti-citrullinated protein antibodies; BMI: body mass index; CRP: C-reactive protein; DAPSA: disease activity index for PsA (range 0-164); DMARD: disease modifying anti-rheumatic drug use past three months; Erosions: reported by radiologist on conventional radiography and/or magnetic resonance imaging; ESR: erythrocyte sedimentation rate; HAQ: health assessment questionnaire (range 0-3); HC: healthy control; LEI: leeds enthesitis index (range 0-6); PASDAS: psoriatic arthritis disease activity score (range 0-10); PASI: psoriasis severity index (range 0-72); PsA: psoriatic arthritis; PsO: psoriasis; RF: rheumatoid factor.*

**Supplemental Table S4.** *Baseline characteristics ELISA cohort – synovial fluid*

| **Characteristic** | **OA**  (n=38) | **Gout**  (n=32) | **PsA**  (n=43) |
| --- | --- | --- | --- |
| Age (years) | 54 ± 10 | 55 ± 12 | 50 ± 12 |
| Female, n (%) * | 17 (45%) | 1 (3%) | 11 (26%) |
| DMARD use, n (%) | 0 (0%) | 0 (0%) | 0 (0%) |
| CRP (mg/L) | NM | NM | 9.5 (0.5-21.3) |
| ESR (mm/hour) | NM | NM | 14 (7-24) |

***Legend****: SF was collected from 2004 to 2014. Presented data are from time of SF collection. Categorical data are presented with frequencies (%) and continuous data are presented as mean ± SD (normally distributed variables) or median (IQR)(non-normally distributed variables). * P value PsA vs. Gout <0.05, and P value OA vs. Gout <0.05.* ***Abbreviations****: CRP: C-reactive protein; DMARD: disease modifying anti-rheumatic drug use past three months; ESR: erythrocyte sedimentation rate; NM: not measured; OA: osteoarthritis; PsA: psoriatic arthritis.*
